# Protective Behavior in Course of the COVID-19 Outbreak—Survey Results From Germany

**DOI:** 10.3389/fpubh.2020.572561

**Published:** 2020-09-24

**Authors:** Daniel Lüdecke, Olaf von dem Knesebeck

**Affiliations:** Institute of Medical Sociology, University Medical Center Hamburg-Eppendorf, Hamburg, Germany

**Keywords:** COVID-19, social inequalities, pandemic, health behavior, educational inequalities, daily practice, sociodemografics factors

## Abstract

**Objective:** The COVID-19 outbreak means far-reaching changes in the organization of daily lives. Disease-related literacy and factors such as age, gender, or education play a major role in shaping individual practices of protective behavior. This paper investigates different types and frequency of practicing protective behaviors, as well as socio-demographic factors that are associated with such behavioral change.

**Methods:** Data stem from a cross-sectional survey in Germany. Three thousand seven hundred and sixty-five people were contacted, 3,186 participated in the survey. Information on behavior to lower the risk of becoming infected with COVID-19 was assessed by nine items (answer options yes/no). For each item, logistic regression models were used to estimate odds ratios (OR), using education, sex, and age as main predictors and adjusting for partnership status and household composition.

**Results:** People with lower educational level were less likely to avoid gatherings (OR = 0.63; 95%CI = 0.48–0.83), adapt their work situation (OR = 0.66; 95%CI = 0.52–0.82), reduce personal contacts and meetings (OR = 0.71; 95%CI = 0.55–0.93), or increase hand hygiene (OR = 0.53; 95%CI = 0.38–0.73). Being female was associated with higher odds of protective behavior for most outcomes. Exceptions were wearing face masks and adapting the own work situation. Associations between respondents' age and individual behavior change were inconsistent and mostly weak.

**Conclusion:** Disease specific knowledge is essential in order to enable people to judge information on COVID-19. Health education programs aiming at improving COVID-19 knowledge are helpful to build up appropriate practices and reduce the spread of the disease. Strategies are needed to guarantee easy access and better dissemination of high-quality news and fact-checks. Socioeconomic characteristics should be taken into account in the development of infection control measures.

## Introduction

Coronavirus disease 2019 (COVID-19) is an illness caused by a novel virus, called Severe Acute Respiratory Syndrome Coronavirus 2 (SARS-CoV-2). It is an emerging respiratory infection that was first discovered in December 2019, in Wuhan city, China (1). Meanwhile, the virus has spread worldwide and the World Health Organization (WHO) officially declared the outbreak as international public health emergency (2). The COVID-19 outbreak has serious impacts on individual behavior as well as the society as a whole and how individuals interact with each other. Due to the extremely high infection rate and relatively high mortality, politics imposed several restrictive measures like social distancing or movement restrictions. Thus, people began worrying about COVID-19 and changed their social behavior accordingly (3). As such, the outbreak implies far-reaching changes in the way they organize their daily lives, e.g., through changes in their working lives and challenges in childcare arrangements. Surveys in different countries have been conducted that investigated the effect of the COVID-19 outbreak on behavior change. For Germany, to the best of our knowledge, despite reports in news and mass media, studies on protective behavior published in scientific journals are still missing. Thus, this paper investigates how the German population deals with the new situation and how individuals change their everyday life.

**Figure 1:**
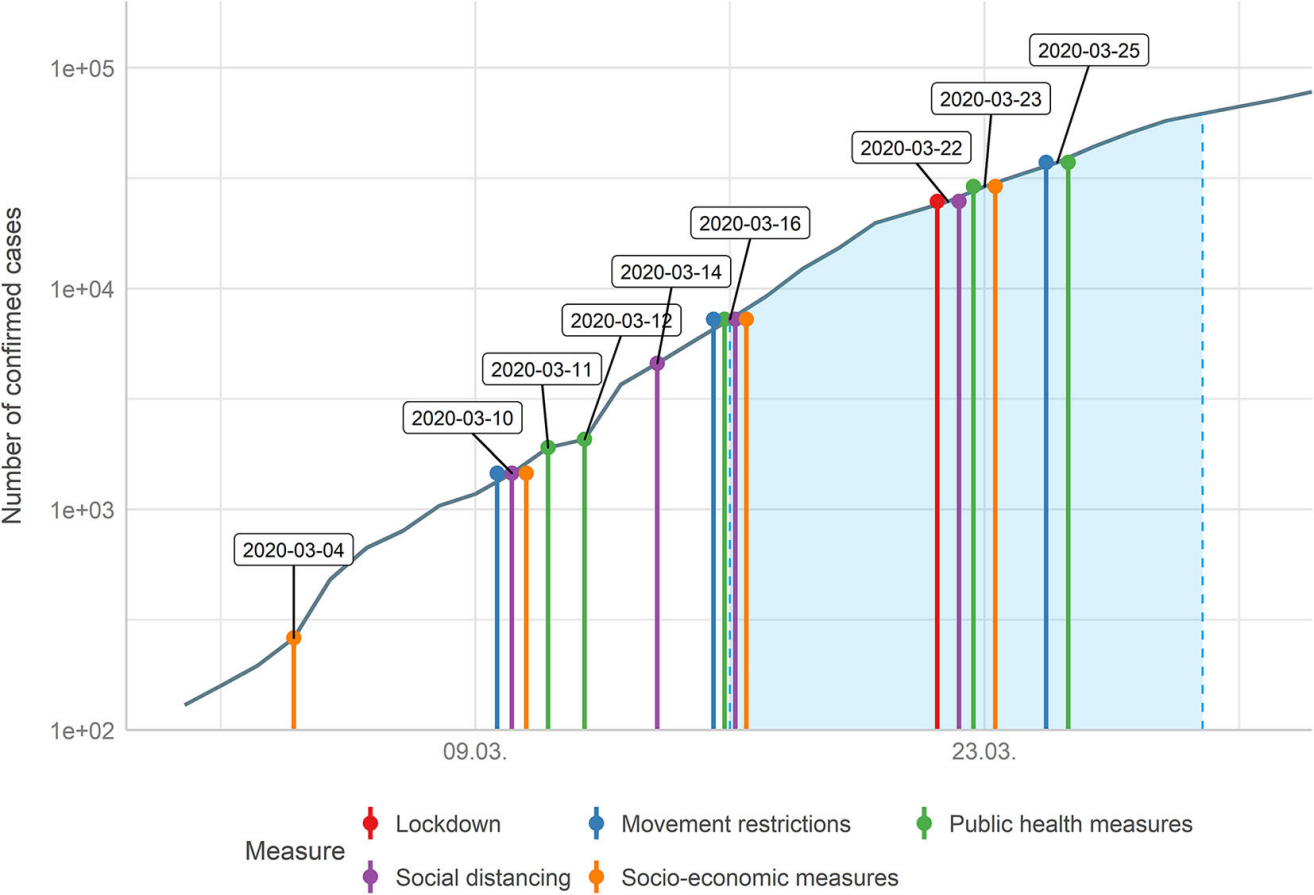
Timeline of measures undertaken in Germany beginning from the first 100 confirmed infectious cases until the end of data collection (end of March 2020, period of data collection highlighted in blue).

Protective behavior and how people organize their daily life depends on public awareness of the threat of the COVID-19 outbreak, which is also influenced by measures like closing borders, bans on gatherings, or movement restrictions. Such preventive and precautionary measures against COVID-19 to control the outbreak were also undertaken in Germany prior to the phase of data collection in this study. Two social distancing measures (all gatherings over 1,000 members, and later all gatherings over 50 members, were canceled), three public health measures (health campaign, special funding for research on coronavirus and isolation/quarantine policies), two movement restriction measures (additional documents required on arrival from certain countries and intensified border controls) and two socio-economic measures (export ban of medical products and suspension of commercial traffic on Sundays) were implemented until the mid of March 2020. On the day when data collection started (16th March), five further measures were undertaken, while seven more measures were introduced during the period of data collection (see Figure 1 and Supplementary Table 1).

In terms of protective behavior, studies have shown that disease-related literacy and factors such as age, gender, or education play a major role in shaping individual practices (4, 5). Against this background, this study tackles following research questions: What kind of protective behaviors have people practiced how often? Which socio-demographic factors are associated with protective behavior in German public's everyday life due to the COVID-19 outbreak?

## Methods

### Study Design and Setting

The data used in this study stem from a special cross-sectional survey from the GESIS panel on the Coronavirus SARS-CoV-2 outbreak in Germany (6). The regular GESIS Panel is a representative probability-based mixed-mode access panel, which started in February 2014. Data collection takes place up to six times a year. Recruitment is based on random samples of individuals from population registries, stratified by regions (7). The complete panel comprises about 5,400 participants aged 18 years and older, of which about three quarter participate online. Data collection was carried out by a German institute for market and social research, Kantar TNS (formerly TNS Infratest).

### Sampling and Participants

Data was collected from 16th to 29th March 2020. Due to the necessity of timely data collection, only the subsample of online respondents was invited to participate in the special survey on the COVID-19 outbreak. Three thousand seven hundred and sixty-five persons were contacted and 3,186 filled out the online-questionnaire (response rate of 84.6%), hence the sample size for this study is *N* = 3,186. Since this special survey represents a subsample of the regular GESIS panel, weights were applied to ensure representativeness. Informed consent for initial participation as well as subsequent panel participation was requested prior to the first interview, and was considered to have been given when individuals have sent back the signed informed consent sheet. All information material and informed consent sheets are available online (8). According to ethical and privacy policies as well as data protection, Kantar TNS follows the high standards of the ICC/ESOMAR International Code on Market, Opinion, and Social Research and Data Analytics (9).

### Additional Data Sources

For Figure 1 and Table 1, we used additional data sources providing information about policy measures and infection numbers. Data on measures undertaken by German politics stem from the acaps-website (10). Data on numbers of confirmed cases of people infected with COVID-19 are taken from the COVID-19 Data Repository by the Center for Systems Science and Engineering (CSSE) at the Johns Hopkins University (11).

**Table 1:** Timeline of measures undertaken in Germany until the end of data collection (end of March 2020).

**Date**	**Category**	**Measure**
2020-02-06	Public health measures	Health campaign.
2020-02-29	Movement restrictions	Additional health/documents requirements upon arrival from certain countries.
2020-03-04	Socio-economic measures	Export ban for medical products.
2020-03-10	Social distancing	All gatherings over 1,000 members are canceled.
2020-03-10	Movement restrictions	Intensification of border controls.
2020-03-10	Socio-economic measures	Suspension on the usual ban of commercial road traffic on Sundays.
2020-03-11	Public health measures	Special funding for research on coronavirus.
2020-03-12	Public health measures	Isolation and quarantine policies.
2020-03-14	Social distancing	All events with more than 50 persons are banned.
2020-03-16	Public health measures	All non-essential surgeries in hospitals will be postponed.
2020-03-16	Movement restrictions	Border closure.
2020-03-16	Social distancing	Schools closure.
2020-03-16	Social distancing	Closure of businesses and public services.
2020-03-16	Socio-economic measures	State of emergency declared.
2020-03-22	Social distancing	Restaurants are being closed; delivery still possible.
2020-03-22	Social distancing	Movement in public spaces is limited to two persons.
2020-03-22	Lockdown	Only essential trips, including for work are still allowed.
2020-03-23	Public health measures	Strengthening the public health system.
2020-03-23	Socio-economic measures	Economic measures.
2020-03-25	Movement restrictions	Seasonal workers and harvesters are banned from entering.
2020-03-25	Public health measures	Financial incentives improved for people to take second job in essential industries, including health.

### Measures

#### Outcome

Information on behavior to lower the risk of becoming infected with COVID-19 was assessed by eight different items: (1) “I have avoided certain (busy) places,” (2) “I have adapted my school or work situation,” (3) “I washed my hands more often and longer,” (4) “I have kept distance to other people (at least 1.5 m),” (5) “I have quarantined myself, although I have no symptoms,” (6) “I used disinfectant,” (7) “I have reduced personal meetings and contacts,” and (8) “I was wearing face masks.” Additionally, one item was used to indicate that no protective behavior was undertaken, “I have taken none of these measures.” All of these items had two answering options, “yes” and “no.”

#### Independent Variables

Educational level was measured using the ISCED-97 scale (12) and was recoded into three categories (low, intermediate, and high educational level). Sex and age were recorded. Age was divided into ten categories, each spanning a range of 5 years, except for the lowest and highest age category (people under 25 and people above 70 years of age, respectively). Legal marital status has four categories: unmarried, married or partnership, widowed, and divorced. Household composition is classified by the total number of household members.

### Statistical Analysis

Descriptive statistics were chosen to document the sample characteristics and the distribution of item categories. Associations between independent variables and binary outcomes were analyzed using logistic regression models. Thus, we calculated nine multiple logistic regression models, each model included the same, complete set of all five independent variables. Odds ratios, 95% confidence intervals, and *p*-values are reported. Multicollinearity was tested for all models. All models had a variance inflation factor (VIF) below 2, indicating no severe collinearity issues (13). Analyses were conducted using the R Statistical Package version 4.0.0 (14).

## Results

### Sample Characteristics

About one third of the respondents had a low, intermediate, or high educational level. Nearly half of the sample were females. Age groups were fairly similarly spread across the sample, varying from 5 to 11% per age group. The major exception was the age group from 51 to 60 years, which accounted for about one quarter of the sample. 42.9% were married or living in partnership, 33.7% were unmarried, 12.9% were divorced, and 10.6% of the respondents were widowed. On average, 1.9 persons (median: 2) lived in a household.

### Measures Undertaken in Germany Related to the COVID-19 Outbreak

To put the results regarding individual behavior and how people change the organization of their daily live into a broader context, measures related to the COVID-19 outbreak undertaken in Germany in the time before and during the survey are described in Figure 1 and Table 1.

Prior to the phase of data collection, two social distancing measures, three public health measures, two movement restriction measures, and two socio-economic measures were conducted. On the day where data collection started (16th March), five further measures were undertaken, while seven more measures were conducted during the period of data collection.

Mandatory face mask wearing in public transport, during shopping, or at weekday markets were introduced after the data collection period.

### Protective Behavior

Table 2 gives an overview of measures of protective behavior. About 88% of the respondents washed their hands more often and longer, while about 82% avoided (busy) places or reduced personal meetings and contacts. Another behavior that most respondents changed was keeping distance to other people (78.5%). These were the measures applied most often. Disinfectants were used by 58% of the sample, while about 39% adapted their school or work situation. Only 10% self-quarantined themselves although having no symptoms, 3% wore face masks and 2.3% took no measures.

**Table 2:** Percentage of protective behavior practiced by respondents (*N* = 3,186, multiple answers possible).

**Protective behavior**	**Answer “Yes,” %**
I washed my hands more often and longer	88.2
I have avoided certain (busy) places	82.3
I have reduced personal meetings and contacts	81.8
I have kept distance to other people (at least 1.5 m)	78.5
I used disinfectant	58.0
I have adapted my school or work situation	39.4
I have quarantined myself, although I have no symptoms	10.1
I was wearing face masks	3.0
I have taken none of these measures	2.3

### Sociodemographic Factors Associated With Protective Behavior

The results from the multiple logistic regression models are presented in Table 3. Compared to individuals with a high education, lower educated persons are less likely to avoid (busy) places (OR = 0.63; 95%CI = 0.48–0.83), to adapt their work situation (OR = 0.66; 95%CI = 0.52–0.82), to increase hand hygiene (OR = 0.53; 95%CI = 0.38–0.73), keep their distance to other people (OR = 0.71; 95%CI = 0.55–0.93), or reduce personal meetings and contacts (OR = 0.57). There is no clear association between lower education and self-quarantining without symptoms, using disinfectants, or wearing face masks.

**Table 3:** Results from the nine multiple logistic regression models on the association of socio-demographic factors and protective behavior.

**Predictors**	**I have avoided certain (busy) places**			**I have adapted my school or work situation**			**I washed my hands more often and longer**		
	**OR**	**95% CI**	***p***	**OR**	**95% CI**	***p***	**OR**	**95% CI**	***p***
(Intercept)	6.52	3.42–12.56	**<0.001**	4.42	2.62–7.52	**<0.001**	6.64	3.09–14.41	**<0.001**
Education (low)	0.63	0.48–0.83	**0.001**	0.66	0.52–0.82	**<0.001**	0.53	0.38–0.73	**<0.001**
Education (middle)	0.85	0.65–1.10	0.212	0.70	0.57–0.86	**0.001**	0.71	0.52–0.96	**0.027**
Female gender	1.47	1.21–1.80	**<0.001**	0.86	0.73–1.01	0.059	2.13	1.67–2.73	**<0.001**
Age	1.03	0.98–1.09	0.190	0.79	0.75–0.82	**<0.001**	1.05	0.99–1.12	0.105
Partnership: single	0.48	0.35–0.65	**<0.001**	0.67	0.52–0.86	**0.002**	0.50	0.34–0.72	**<0.001**
Partnership: divorced	1.00	0.70–1.44	0.981	1.30	1.00–1.69	0.051	0.52	0.36–0.77	**0.001**
Partnership: widowed	0.93	0.63–1.38	0.698	0.81	0.59–1.09	0.165	1.08	0.65–1.86	0.774
Size of household	0.88	0.75–1.04	0.122	1.02	0.89–1.17	0.740	1.10	0.90–1.34	0.361
**Predictors**	**I have kept distance to other people (at least 1.5 m)**			**I have quarantined myself, although I have no symptoms**			**I used disinfectant**		
	**OR**	**95% CI**	***p***	**OR**	**95% CI**	***p***	**OR**	**95% CI**	***p***
(Intercept)	2.05	1.13–3.72	**0.018**	0.16	0.07–0.36	**<0.001**	1.37	0.82–2.29	0.226
Education (low)	0.71	0.55–0.93	**0.013**	1.13	0.80–1.59	0.485	1.12	0.90–1.39	0.313
Education (middle)	0.64	0.51–0.81	**<0.001**	0.78	0.56–1.08	0.133	1.16	0.96–1.42	0.132
Female gender	1.58	1.31–1.90	**<0.001**	1.24	0.97–1.60	0.092	1.40	1.20–1.63	**<0.001**
Age	1.13	1.07–1.18	**<0.001**	0.91	0.85–0.97	**0.004**	0.92	0.88–0.96	**<0.001**
Partnership: single	0.63	0.47–0.85	**0.002**	1.30	0.88–1.93	0.195	0.89	0.70–1.14	0.356
Partnership: divorced	0.70	0.52–0.97	**0.028**	2.10	1.41–3.09	**<0.001**	0.90	0.70–1.17	0.431
Partnership: widowed	0.54	0.39–0.76	**<0.001**	1.56	0.97–2.44	0.059	0.79	0.60–1.03	0.078
Size of household	1.08	0.93–1.26	0.307	0.94	0.76–1.15	0.529	1.22	1.07–1.39	**0.003**
**Predictors**	**I have reduced personal meetings and contacts**			**I was wearing face masks**			**I have taken none of these measures**		
	**OR**	**95% CI**	***p***	**OR**	**95% CI**	***p***	**OR**	**95% CI**	***p***
(Intercept)	1.76	0.94–3.31	0.078	0.02	0.00–0.10	<0.001	0.02	0.00–0.12	<0.001
Education (low)	0.57	0.43–0.75	**<0.001**	1.28	0.67–2.47	0.459	2.03	1.03–4.08	**0.044**
Education (middle)	0.75	0.58–0.97	**0.027**	1.41	0.79–2.58	0.256	0.77	0.35–1.65	0.511
Female gender	1.70	1.40–2.08	**<0.001**	1.01	0.65–1.57	0.950	0.49	0.28–0.83	**0.010**
Age	1.15	1.09–1.21	**<0.001**	1.03	0.92–1.16	0.621	0.97	0.85–1.10	0.624
Partnership: single	0.78	0.58–1.06	0.118	0.66	0.31–1.38	0.278	1.14	0.51–2.50	0.742
Partnership: divorced	0.71	0.52–0.99	**0.040**	1.37	0.71–2.54	0.332	0.96	0.36–2.25	0.933
Partnership: widowed	0.89	0.60–1.33	0.544	0.48	0.17–1.14	0.125	1.15	0.40–2.82	0.777
Size of household	1.15	0.98–1.35	0.092	1.01	0.68–1.48	0.958	1.12	0.74–1.70	0.585

*Shown are odds ratios (OR), 95% confidence intervals (CI) and p-value (p) (N = 3,186). Significant p-values (p < 0.05) in bold*.

Female participants are more likely to avoid (busy) places (OR = 1.47; 95%CI = 1.21–1.80), increase hand hygiene (OR = 2.13; 95%CI = 1.67–2.73), keep their distance to other people (OR = 1.58; 95%CI = 1.31–1.90), use disinfectants (OR = 1.40; 95%CI = 1.20–1.63), or reduce personal meetings and contacts (OR = 1.70; 95%CI = 1.40–2.08). Compared to male participants, female respondents are less likely to adapt their work situation (OR = 0.86; 95%CI = 0.73–1.01) and more likely to self-quarantine (although having no symptoms, OR = 1.24; 95%CI = 0.97–1.60). However, evidence for these associations is less pronounced due to missing statistical significance.

Age shows inconsistent patterns across the nine different behavior change outcomes. Older persons are less likely to have adapted their work situation (OR = 0.79; 95%CI = 0.75–0.82), to self-quarantine (OR = 0.91; 95%CI = 0.85–0.97) or to use disinfectants (OR = 0.92; 95%CI = 0.88–0.96). Probability of behavior change increased with older age for keeping distance to other people (OR = 1.13; 95%CI = 1.07–1.18) and reducing personal meetings and contacts (OR = 1.15; 95%CI = 1.09–1.21). No clear associations were found related to the measures avoiding (busy) places, increased hand hygiene, and wearing face masks.

Compared to persons living in partnership, single persons were less likely to avoid (busy) places (OR = 0.48; 95%CI = 0.35–0.65) or to adapt their work situation (OR = 0.67; 95%CI = 0.52–0.86). The odds for both single and divorced persons were lower for hand hygiene (OR = 0.50; 95%CI = 0.34–0.72 and OR = 0.52; 95%CI = 0.36–0.77) and to keep distance to other people (OR = 0.63; 95%CI = 0.47–0.85 and OR = 0.70; 95%CI = 0.52–0.97). Divorced persons were more likely to self-quarantine although not having symptoms (OR = 2.10; 95%CI = 1.41–3.09) and less likely to reduce personal contacts and meetings (OR = 0.71; 95%CI = 0.52–0.99).

For most behavioral change outcomes, we found no clear association with the size of the household. The only statistically significant relation was found for using disinfectants (OR = 1.22; 95%CI = 1.07–1.39).

### Sociodemographic Factors Associated With Taking no Protective Behavior

Lower education is associated with higher odds of taking no measures at all (OR = 2.03; 95%CI = 1.03–4.08). The odds of taking no measure at all are considerably lower for female persons (OR = 0.49; 95%CI = 0.28–0.83), as compared to male persons. We found no clear evidence in our data for or against taking no measures regarding participants' age (OR = 0.97; 95%CI = 0.85–1.10). The same holds true for the different types of partnership status as well as household size.

## Discussion

The study reported in this paper sought to understand those socio-demographic factors that are associated with behavior change in everyday life due to the COVID-19 outbreak in the German population.

### Summary of Main Findings

One major finding is the strong and consistent association of lower educational level with most of the protective behavior outcomes. Interestingly, while high education seems to be clearly associated with behavior change, we found no educational gradient, i.e., there is no consistent pattern across all eight outcomes indicating a positive linear relationship between education and protective behavior. However, for most protective behaviors such as avoid gatherings, reduce personal contacts and meetings, or increased hand hygiene, our results suggest that lower educated people are less likely to undertake these measures. Contrary, lower education is associated with higher odds for undertaking no protective behavior at all. This is in line with surveys from other countries. A cross-sectional study among adults in the United States found a gradient between health literacy and change of daily routines (15). Accordingly, people with higher health literacy were more likely to change their behavior. A study among adults who were studying at a medical university or completed their medical education showed that both educational attainment and medical education is associated with protective behavior. Higher educated people adopted more preventive measures like wearing masks or using disinfectants (16). Another recent study from Saudi Arabia reported similar results, i.e., higher educated participants were more likely to adopt protective practices (17).

A second major finding is the association between gender and protective behavior. Being female was associated with higher odds of protective behavior for most outcomes. Exceptions were wearing face masks, which was not clearly associated with male or female persons, and adapting the school or work situation, where female respondents were less likely to do so. A higher willingness to change their behavior and to act preventively seems to be more common among women than among men in Germany. This result is also in line with other studies (17).

A third and rather surprising finding is the inconsistent and mostly weak association between individual behavior change and the respondents' age. Against the background that higher age is a known risk factor for a more severe COVID-19-associated illness and death (18, 19), which has also been widely reported in media, we would have assumed that higher age groups show more protective behavior.

Gender and education are important socio-demographic factors related to protective behavior. It is known that there is a socioeconomic gradient, resulting in social and health inequalities related to the COVID-19 outbreak (20, 21). In particular, socioeconomic characteristics like lower income and lower education are associated with an increased risk of COVID-19 related mortality (22, 23), which stresses the necessity to reduce health inequalities (24). Similarly, lower educated people almost have a doubled risk of getting infected with COVID-19 as compared to higher educated people (25). There are three possible explanations for the socioeconomic and educational disparities discussed in the literature. Differential exposure, differential susceptibility, and differential access to health care (26, 27). Differential exposure refers to different living and working conditions. With exception of people working in critical jobs (like health care workers), lower educated people less often have the opportunity for home office and are more likely to have jobs with higher risk exposure (28). Differential susceptibility describes the correlation between higher morbidity and lower income and education. People of lower status groups have disproportionately higher levels of health conditions, that increase the risk of complications from COVID-19 (29). Differential access to health care means that people with lower income and lower education face more barriers in accessing health care provision, are less likely to use preventive services and health services and take more time before seeking help (30–33). Moreover, protective behavior and the perception of health risks varies by socio-demographic factors and socioeconomic status. For instance, people with lower education underestimate the cancer risk due to smoking (34, 35). Thus, differential perception of health risks or differential perception the usefulness of protective behavior would be another explanation for the differences between respondents depending on socio-demographic factors.

In conclusion, adopting or failing to take protective behavior is not only related to sufficient knowledge about COVID-19. People might not have the possibility to change working or living conditions in order to better practice protective behavior. Hence, it is important to either provide sufficient opportunities for home office, or public health measures should aim at making workplace conditions more secure in accordance with distancing or hygiene measures. Another important aspect is the availability and affordability of necessary equipment like face masks or disinfectants. For instance, at the beginning of the pandemic in Germany, the government did not suggest wearing face masks simply for the reason that those masks were not available for the broader public. Furthermore, lower education is often associated with lower health literacy (36), which also affects how people are able to cope with the enormous amount of information on COVID-19 in the media, and how to separate useful and important information from misinformation. A recent study among the German population showed that 56% of the respondents were unsettled by the flood of information, and only 51% believed themselves capable of judging whether information about COVID-19 was trustworthy (37). A lack of knowledge how to properly adopt protective behavior may result in lower educated people being more susceptible to infection risks. As such, improving disease specific knowledge and health literacy and developing strategies for dealing with misinformation is essential.

### Strengths and Limitations

One strength of this study is the relatively up-to-date data, which comes from a representative population-based survey. This allows to draw generalized conclusions about protective behavior in the German population and to identify factors that are related to individual behavior change until the end of March 2020. Another strength is the amount of different behaviors that were assessed. Other studies partially only assessed the numbers of protective practices or data was less detailed relating to the different measures (17, 38).

This study also has some limitations. Since protective behavior may depend on peoples' health status, one limitation of this survey is the lack of information on this aspect. We cannot rule out that part of the explanation why some people tend to adopt protective practices more intensively than other people might be due to their increased health risks. Furthermore, although we have educational status as an indicator for a person's socioeconomic status, the survey collected no data on income. This partly restricts comparisons of our results with other studies that found associations of socioeconomic position and COVID-19 related protective behavior. Another limitation is that the data is cross-sectional and stems from an early stage of the COVID-19 outbreak in Germany. Hence, we cannot predict how people would change their protective behavior during the later course of the pandemic, which would require a longitudinal survey. Finally, the survey questions assessed protective behavior only with a binary yes/no measure. Although this allows to analyze whether or not people have changed their behavior to response to the pandemic, it limits conclusions about the extent to which people have adopted protective practices. Moreover, these questions provide insufficient information about the perception of the different behaviors and measures like “social distancing.” It may be that people with certain socio-demographic background attribute a different importance to these behaviors and measures.

## Conclusion

Disease specific knowledge is essential in order to enable people to judge information on COVID-19 and to motivate people to change their behavior. Health education programs aiming at improving COVID-19 knowledge can be helpful to build up appropriate practices and thereby reduce the spread of the disease. Socioeconomic characteristics should be taken into account in the development of infection control measures like target-group oriented communication or needs-based social and financial support services. Future research on COVID-19 should take socioeconomic characteristics into account to better determine risk groups in order to work toward greater equity in health.

## Data Availability Statement

The datasets presented in this article are not readily available because data is available as public-use file for free upon request. Requests to access the datasets should be directed to https://www.gesis.org/gesis-panel/coronavirus-outbreak/.

## Ethics Statement

Ethical review and approval was not required for the study on human participants in accordance with the local legislation and institutional requirements. The patients/participants provided their written informed consent to participate in this study.

## Author Contributions

DL and OK developed the research questions. DL prepared, analyzed and interpreted the data, and drafted and finalized the manuscript. OK substantially contributed to interpreting the data, drafting the manuscript, and critically revised and approved the final manuscript. All authors contributed to the article and approved the submitted version.

## Conflict of Interest

The authors declare that the research was conducted in the absence of any commercial or financial relationships that could be construed as a potential conflict of interest.
