# Supplementary material for: Protective Behavior in Course of the COVID-19 Outbreak—Survey Results From Germany
Source: Front Public Health. 2020 Sep 24;8:572561. doi: 10.3389/fpubh.2020.572561 (PMC7543680; doi:10.3389/fpubh.2020.572561)
Supplement: Supplementary file 1 [file Table_1.DOCX]

Supplementary Material

# Supplementary Tables

**Supplementary Table 1**: Sample Characteristics, N=3,186

| *Characteristic* | *Weighted* | *Unweighted* |
| --- | --- | --- |
| Education (low), % | 33.4 | 11.2 |
| Education (middle), % | 31.7 | 30.7 |
| Education (high), % | 34.9 | 58.1 |
| Female gender, % | 50.7 | 48.7 |
| Age (< 26 years), % | 8.1 | 2.8 |
| Age (26-30 yrs), % | 5.2 | 7.1 |
| Age (31-35 yrs), % | 7.6 | 7.3 |
| Age (36-40 yrs), % | 9.7 | 8.7 |
| Age (41-45 yrs), % | 4.8 | 8.4 |
| Age (46-50 yrs), % | 6.4 | 9.7 |
| Age (51-60 yrs), % | 24.0 | 26.0 |
| Age (61-65 yrs), % | 8.3 | 10.3 |
| Age (66-70 yrs), % | 11.4 | 9.5 |
| Age (> 70 yrs), % | 14.6 | 10.1 |
| Partnership (married), % | 42.9 | 63.6 |
| Partnership (single), % | 33.7 | 25.2 |
| Partnership (divorced), % | 12.9 | 7.7 |
| Partnership (widowed), % | 10.6 | 3.4 |
| Mean Household Size (and SD) | 1.9 (0.8) | 2.3 (0.7) |
